# Comparative Accuracy of Dental Age Estimation Methods in Adolescents and Young Adults: A Systematic Review With Cameriere’s Method as the Reference Standard

**DOI:** 10.7759/cureus.100563

**Published:** 2026-01-01

**Authors:** Azhar A Khan, Sumit Bhateja

**Affiliations:** 1 Department of Oral Pathology, School of Dental Sciences, Manav Rachna International Institute of Research and Studies, Faridabad, IND; 2 Department of Oral Pathology and Microbiology, Manav Rachna Dental College, Faridabad, IND; 3 Department of Oral Medicine and Radiology, School of Dental Sciences, Manav Rachna International Institute of Research and Studies, Faridabad, IND

**Keywords:** cameriere’s third molar maturity index, dental age, newcastle–ottawa scale, pulp-to-tooth area ratio, tooth coronal index

## Abstract

Dental age estimation is a crucial tool in forensic science, particularly for legal purposes where age verification is essential. This process helps distinguish minors from adults, influencing decisions in criminal, immigration, and civil matters. The Cameriere’s Third Molar Maturity Index (I3M) is one of the reliable techniques for the assessment of age.

After being retrieved from databases such as PubMed, Scopus, the Cochrane Library, and EBSCOhost, publications from 2013 to 2025 were screened against our inclusion criteria. As a result, five articles were included in the systematic review. The Newcastle-Ottawa scale (NOS) is used for the assessment of risk of bias of included studies, and meta-analysis was not possible due to the heterogeneity of selected studies in terms of methods of analysis.

Results from the studies reviewed indicate that Cameriere’s I3M is the most dependable method for dental age estimation in adolescents and young adults. Evidence from comparative analyses shows that I3M performs better than the London Atlas for individuals aged 14-22, and additional findings support its effectiveness across similar age ranges. Assessments based on root pulp visibility have also been reported to provide slightly higher accuracy in the 15-22 age group. Overall, the systematic review confirms that Cameriere’s I3M remains the most reliable approach for estimating dental age in adolescents and young adults.

## Introduction and background

Dental age estimation is a critical aspect of forensic science, especially in situations where the age of an individual cannot be verified through reliable documentation. This process is particularly important in legal cases involving immigration, criminal responsibility, or civil rights, where distinguishing between minors and adults significantly influences judicial outcomes [1-4]. For instance, criminal penalties, eligibility for asylum, and guardianship decisions often depend on whether an individual has reached the age of legal majority [5,6].

Teeth, especially the third molars, or wisdom teeth, are frequently used for age estimation due to their relatively predictable development and resistance to environmental influences such as malnutrition or systemic disease. Third molars continue to develop into the late teenage years and early twenties, making them valuable indicators of age during adolescence and early adulthood [7-9].

Several radiographic methods have been developed for dental age estimation, each based on specific aspects of tooth development. One of the most widely used techniques is Cameriere’s Third Molar Maturity Index (I3M), which evaluates the degree of root development and open apices in third molars using panoramic radiographs [10,11]. This method is particularly useful in individuals aged between 15 and 25 years and has shown high accuracy in determining whether a person has reached 18 years of age, the typical legal threshold for adulthood in many countries [12].

Demirjian’s method is another commonly used approach, which assigns developmental stages to teeth based on radiographic appearance. While it is considered highly accurate in children and early adolescents, its effectiveness declines in older age groups as dental maturation nears completion [13,14]. Olze’s method, based on the visibility of the pulp in third molars, is used primarily in older adolescents and young adults but has shown variability across populations. The London Atlas provides a visual guide to dental development stages; however, it is generally considered less precise for determining legal adulthood compared to methods like I3M [15,16].

Dental age estimation remains an essential forensic tool, particularly for young individuals. Among available techniques, Cameriere’s I3M stands out for its reliability and simplicity. However, the choice of method should be tailored to the individual’s age range, population background, and the legal context of the case [17,18].

## Review

Materials and methods

Protocol Registration

**Figure 1:**
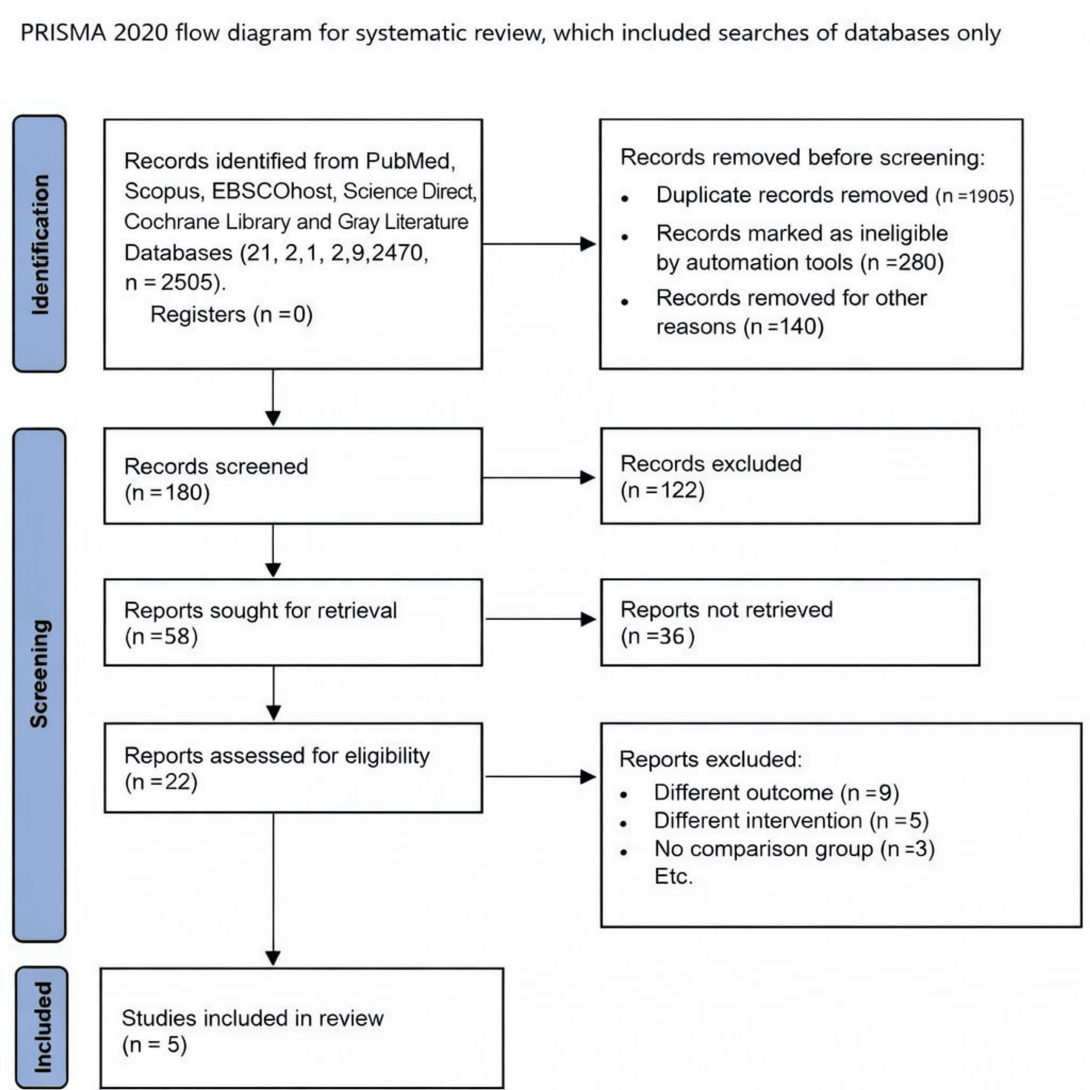
PRISMA 2020 flow diagram for systematic review, which included searches of databases only The tools used in this study are free to use [19,20]. PRISMA: Preferred Reporting Items for Systematic Reviews and Meta-Analyses

This systematic review adhered to the Population, Intervention, Comparator, Outcome, and Study (PICOS) design criteria, with a current search ensuring no prior published systematic reviews on related subjects. The Preferred Reporting Items for Systematic Reviews and Meta-Analyses (PRISMA) 2020 was considered in this systematic review (Figure 1). This review used the Cochrane Handbook for Systematic Reviews of Interventions as a guide, and the study protocol was registered in the Open Science Framework (OSF) [19,20].

Focused Question

Using the PICOS framework, this review question was generated using Population (P): radiograph of adolescents and adults aged 18 years or young adults; Intervention (I): various dental age estimation methods; Comparison (C): Cameriere’s method for dental age estimation; ‘‘accuracy” in estimating dental age was used to measure Outcome (O). Research (S) accuracy of various radiographic dental age estimation methods compared to Cameriere’s method for dental age estimation. Through mutual consent, a group of authors (AK and SB) designed the research question by taking into account the need for this systematic review. As a result, this review compares the evaluation of the accuracy of various dental age estimation methods in adolescents and young adults and includes observational, cross-sectional, and cohort studies. Thus, the research question is, “Is the accuracy of the Camerier method for dental age estimation more effective in young adults when compared with various other dental age estimation methods?”

Literature Search Strategies

Scopus, PubMed, EBSCOhost, and Cochrane Library were included as major search engines for article retrieval. The search strategy comprised text words and MeSH terms, which consist of the following keywords: “Dental age estimation methods,” “Accuracy,” and “Adult population” and Cameriere and the following combination of search terms were used to search articles: (Dental age estimation methods) AND (Accuracy) AND (Adult population OR ‘‘Young population”) AND (Cameriere) using the All Fields function in PubMed, Scopus, EBSCOhost, Science Direct, Google Scholar, the Title-ABS-Key function in Scopus, and the Advance Search function with a Medical subheading (MeSH) in the Cochrane Library database. Articles were initially filtered according to the title and abstract, and papers that matched the inclusion criteria were considered relevant.

Inclusion Criteria

Included studies were cross-sectional, cohort, or observational, involving individuals aged 18 or young adults. They used radiographic images for dental age estimation and compared Cameriere’s I3M method with other techniques like Demirjian’s stages or the London Atlas. Studies reporting accuracy, sensitivity, specificity, or error rates were considered eligible.

Exclusion Criteria

Studies with individuals younger than 18 years or animal studies were excluded. Studies that do not apply the Cameriere method with other methods for dental age estimation and do not focus on accuracy, sensitivity, specificity, or other relevant metrics for age estimation, such as a small amount of radiographic data, were also not included in the study.

Quality Assessment of Included Studies

Two assessors, AK and SB, independently assessed the risk of bias using the Newcastle-Ottawa scale (NOS) for each included study as per the PRISMA 2020 statement.

Data Collection/Extraction Process

AK and SB extracted the data independently, reviewed and finalized the data selection, and resolved disagreements through discussion. Data relevant to the inclusion criteria were retrieved and otherwise excluded. The data accuracy was confirmed by both authors.

Results

The initial database identified 2,505 studies. After examination of titles, abstracts, and duplicate elimination, reviewers excluded 2500 studies that did not meet the eligibility criteria. The remaining five studies [1,5,21-23] were included in this systematic review, focusing on the accuracy of the Camerier method for dental age estimation. These studies specifically compared the Cameriere method with other dental age estimation techniques in young adults to assess their relative effectiveness.

Study Characteristics

This systematic review included five studies, as shown in Table 1, that compared different methods for dental age estimation. Akman et al. (2022) found that in Turkish individuals aged 14 to 21, both Demirjian's stages and Cameriere's I3M were more accurate than the London Atlas method. Hatice et al. (2017) reported that Drusini's method was not effective in the Turkish population, while Cameriere's method provided a more accurate estimation. Kumar et al. (2019) found that Olze et al.'s stages of root pulp visibility were more accurate than I3M in Indian individuals aged between 15 and 22. In Brazil, Correia et al. (2020) concluded that both the London Atlas and I3M methods had similar accuracy in estimating dental age for individuals aged between 16 and 21. Lastly, Campos et al. (2020) observed that while the Kvaal method was more accurate for individuals aged between 20 and 39, the Cameriere method was superior for those over 40 years [1,5,21-23].

**Table 1:** Outcomes of studies included in the systematic review The systematic review included the outcomes of the studies, which summarized populations, radiographic methods, dental age estimation techniques, Cameriere's methods, and the accuracy and conclusions reported in each study. Cameriere's I3M: Cameriere's Third Molar Maturity Index

Reference/Journal	Population/Zone/Age	Radiographic Methods of Study	Various Dental Age Estimation Methods	Cameriere’s Age Estimation Methods	Accuracy of Age Estimation	Conclusion
Hatice BD et al. Dentomaxillofac Radiol. (2017) [1].	200 Turkish individuals aged 20 to 75 years old	Panoramic radiograms	Drusini's	Cameriere’s	Accuracy of age estimation	Drusini's method isn't effective for estimating age in the Turkish population. However, using Cameriere's method, a more accurate age estimation model was developed.
Akman H et al. Clin Oral Investig. (2022) [5].	500 Turkish individuals aged 14-22 years	Panoramic radiograms	Demirjian's maturation stages and The London Atlas	Cameriere's third molar maturity index	Accuracy of age estimation	Demirjian's stages and I3M methods were more accurate than the London Atlas method for estimating adulthood, with the difference being statistically significant (p < 0.05).
Correia AM et al. Forensic Sci Int. (2020) [21].	1200 Northeastern Brazil individuals from 16 to 21 years	Panoramic radiographs	London Atas technique and I3M	Cameriere's third molar maturity index	Accuracy of age estimation	Both methods were equally accurate in terms of the estimation of dental age.
Joyce Campos et al. Braz Oral Res. (2020) [22].	320 Brazilian individuals from age groups of 20-29 and 30-39 years, and for those over 40 years	Periapical radiographs	Kvaal’s	Cameriere’s methods (canine)	Accuracy of age estimation	The Kvaal method was more accurate for the age groups of 20-29 and 30-39 years, and for those over 40 years, the Cameriere method was the most accurate.
Kumar GK et al. J Forensic Dent Sci. (2019) [23].	615 Indian individuals from 15 to 22 years	Digital orthopantomographs (15-22)	Olze et al., stages of radiographic visibility of root pulp	Cameriere's third molar maturity index	Accuracy of age estimation	Stage 0 of Olze's radiographic root pulp visibility was found to be more accurate than the cutoff value of I3M.

Characteristics of Individual Studies

Hatice BD et al. (2017) assessed Drusini’s and Cameriere’s methods for age estimation in a Turkish cohort using panoramic radiographs from 200 individuals. The Tooth Coronal Index (TCI) showed weak correlation with age, rendering Drusini’s method unreliable. Conversely, the Pulp-to-Tooth Area Ratio (AR) of the maxillary canine exhibited a strong negative correlation (r = -0.716). A regression model based on AR explained 51.2% of age variance with a 9.23-year standard error and demonstrated satisfactory accuracy, supporting Cameriere’s method as effective for forensic age estimation in this population [1].

The study conducted by Akman H et al. in 2022 aimed to test the three methods, namely Cameriere’s method, Demirjian’s maturation stages, and the London Atlas. The researchers used panoramic X-rays from 500 Turkish individuals aged 14 to 21. The findings showed that I3M and Demirjian’s stages were more accurate than the London Atlas in distinguishing between adults and minors. Statistical analysis also revealed that the methods and the person’s sex significantly influenced the results. In conclusion, Cameriere’s I3M and Demirjian’s stages were reliable for determining adult status. These methods are particularly useful in legal and forensic contexts, such as assessing age for legal purposes or criminal investigations [5].

Correia AM et al. evaluated the London Atlas and Cameriere’s I3M methods for distinguishing adult status in a sample of 1,200 individuals aged 16 to 21 years from Northeast Brazil. Both methods demonstrated high sensitivity (I3M: 94.1%, London Atlas: 92.3%) and similar overall accuracy (~79.8%), but exhibited low specificity (I3M: 55.4%, London Atlas: 56%), indicating a risk of misclassifying minors as adults. These results suggest that although effective for dental age estimation, both methods require refinement to reduce false positives and enhance legal reliability [21].

Campos et al. (2020) compared the accuracy of Kvaal’s and Cameriere’s methods for age estimation in 320 Brazilian adults using periapical radiographs. Kvaal’s method showed greater accuracy in individuals aged 20-39 years, with smaller discrepancies between chronological and estimated ages. Cameriere’s method was more precise in the 40-to-59 age group. Both techniques were simple, noninvasive, and demonstrated reproducibility. The study concluded that Kvaal’s method is preferable for younger adults, whereas Cameriere’s method is more suitable for older adults, with both providing reliable age estimates [22].

Kumar GK et al. compared Olze et al.'s stages and Cameriere’s method (I3M < 0.08) for estimating the age of majority in Indian individuals. Analyzing 615 digital orthopantomographs of individuals aged 15 to 22, the study evaluated sensitivity, specificity, and likelihood ratios. Results showed that Olze’s Stage 0 method had higher sensitivity and specificity (0.72-0.91) compared to I3M, which had moderate sensitivity (0.67-0.76) and specificity (0.72-0.76). Olze’s method also had better positive likelihood ratios (5.18 for males, 8.63 for females), indicating greater accuracy. In conclusion, Olze’s Stage 0 method was more accurate than I3M for distinguishing adults from minors, making it a more reliable tool for age estimation in legal contexts [23].

Modified Newcastle-Ottawa Scale for Risk of Bias Assessment in Dental Age Estimation Studies

The risk of bias in the included studies was evaluated using a modified version of the NOS as shown in Table 2, specifically adapted for dental age estimation research. The assessment was performed across three domains: Selection (maximum 4 stars), Comparability (maximum 2 stars), and Outcome (maximum 3 stars), yielding a total maximum score of 9. Studies scoring 7-9 were categorized as low risk of bias, 4-6 as moderate risk, and ≤3 as high risk, as mentioned in Table 2. The quality assessment of the selected studies using the adapted NOS indicates that most of the research demonstrates a low risk of bias. Studies by Akman et al. (2022), Kumar et al. (2019), and Correia et al. (2020) exhibited high methodological rigor, with well-defined sample populations, appropriate use of radiographic techniques, and thorough statistical analyses. These factors contribute to the reliability of their findings in dental age estimation [24,25].

**Table 2:** Risk of bias assessment using the modified Newcastle-Ottawa scale The tools used in this study are free to use [24,25].

Study	Selection (Max 4 Stars)	Comparability (Max 2 Stars)	Outcome (Max 3 Stars)	Total Score (/9)	Risk of Bias
Akman H et al. (2022) [5]	★★★★ (Large sample, clear population, valid radiographic method, inclusion criteria defined)	★★ (Compared methods within the same population)	★★ (Statistical significance, appropriate method)	8	Low
Hatice BD et al. (2017) [1]	★★★ (Small sample, clear population, valid method)	★ (Limited comparability; only one method used)	★★ (Clear reporting, limited method reliability)	6	Moderate
Kumar GK et al. (2019) [23]	★★★★ (Large sample, narrow age range, valid technique)	★★ (Compared two methods effectively)	★★ (Strong results, clear outcomes)	8	Low
Correia AM et al. (2020) [21]	★★★★ (Very large sample, well-defined criteria)	★★ (Compared two methods with equal accuracy)	★★ (Good analysis and outcome description)	8	Low
Joyce Campos et al. (2020) [22]	★★★ (Moderate sample, multiple age groups, defined methods)	★★ (Methods compared across age brackets)	★★ (Clear outcome, age-specific accuracy)	7	Low

While the study by Hatice et al. (2017) showed a moderate risk of bias due to a relatively small sample size and limited method comparison, it still provided valuable insights. Campos et al. (2020) also demonstrated good methodological quality, although some limitations were noted in terms of sample representation across age groups. Across the studies, Cameriere’s method, particularly the I3M, consistently showed strong performance and accuracy in estimating chronological age. It was frequently found to be more precise than alternative methods such as Demirjian’s stages, the London Atlas, Drusini’s, and Kvaal’s techniques. These findings support the application of Cameriere’s method as a reliable tool in forensic dental age estimation, especially when supported by a robust study design and statistical validation [26].

Discussion

Dental age estimation plays a critical role in forensic science, particularly in distinguishing minors from adults within legal contexts. Various methods have been developed, including Cameriere’s I3M, Demirjian’s maturation stages, Olze et al.’s root pulp visibility method, the London Atlas, and Drusini’s method, each differing in accuracy depending on the population and age group assessed.

Cameriere’s I3M Method: A Reliable and Versatile Approach

Among these, Cameriere’s I3M method is widely recognized for its reliability and versatility. This technique assesses the degree of third molar development using panoramic radiographs, offering a straightforward, reproducible, and objective approach suitable across a broad age range. Studies conducted in various populations, including Turkish and Indian cohorts, have demonstrated the efficacy of Cameriere’s I3M in accurately identifying individuals aged 18 years and older (Akman et al., 2022; Kumar et al., 2019) [5,23]. Its applicability is particularly valuable in forensic settings where establishing legal adulthood is necessary for criminal liability, immigration, or other legal decisions.

The simplicity of Cameriere’s I3M is a notable advantage; it requires only accessible, noninvasive panoramic radiographs without complex analytical tools or specialized equipment. The method has a strong correlation with chronological age across diverse populations, underlining its robustness. For instance, Hatice et al. (2017) reported superior performance of Cameriere’s I3M over Drusini’s method in a Turkish population, where the latter showed weak age correlations [1]. Additionally, Cameriere’s method maintains accuracy not only in adults over 18 but also in younger adults, providing a practical alternative to methods like Demirjian’s, which tend to decline in reliability beyond adolescence.

Demirjian’s Maturation Stages: The Gold Standard for Youth

Demirjian’s maturation stages remain the gold standard for dental age estimation in children and adolescents. This method evaluates the development of the mandibular third molar and other teeth, and it has been validated across diverse populations. Its accuracy in estimating age, particularly in adolescents approaching the age of majority, is well documented (Akman et al., 2022) [5]. However, as individuals transition into adulthood, the precision of Demirjian’s stages diminishes due to slower and less distinguishable tooth development, reducing its effectiveness for older age groups.

Moreover, Demirjian’s method involves detailed staging of tooth formation, which can be time-consuming compared to Cameriere’s I3M, which offers quicker and simpler assessment. Consequently, while Demirjian’s stages are invaluable for younger populations, their applicability in adult forensic age estimation is limited when high precision is required.

Olze’s Root Pulp Visibility Stages: Population-Specific Utility

Olze's root pulp visibility method assesses the development and visibility of the pulp chamber, with specific stages correlating to age. Some studies indicate that this method can surpass Cameriere’s I3M in accuracy within certain populations. For example, research in Hyderabad revealed that Stage 0 of pulp visibility, when the pulp chamber is fully visible, provided more accurate age estimates than Cameriere’s index (Kumar et al., 2019) [23]. However, Olze’s method lacks extensive validation across diverse populations and is not universally applicable in forensic contexts. This limitation confines its routine use primarily to specific populations where dental development patterns align with the method’s assumptions.

The London Atlas: A Practical But Less Precise Tool

The London Atlas, which categorizes third molar development into standardized stages, offers another age estimation option. Although it is widely used, evidence suggests it is less accurate than Cameriere’s I3M and Demirjian’s stages, especially when precise age determination is critical. Correia et al. (2020) compared the London Atlas and Cameriere’s method in a Brazilian population, finding similar overall accuracy but greater reliability of Cameriere’s I3M for distinguishing legal adulthood (age ≥ 18 years) [21]. Despite its lower precision, the London Atlas remains useful for general dental maturity assessment in routine forensic practice, particularly when detailed age estimates are not essential.

Drusini’s Method: Limited Forensic Application

Drusini’s method estimates age by analyzing the ratio between the coronal pulp cavity and crown height. However, this approach has shown limited effectiveness in some populations. Hatice et al. (2017) demonstrated weak correlations between Drusini’s measurements and chronological age in a Turkish sample, indicating poor applicability [1]. Given these limitations and the availability of more accurate methods, such as Cameriere’s I3M, Drusini’s method is not recommended for standard forensic age estimation.

The modified NOS effectively assesses the methodological quality and risk of bias in dental age estimation studies. Most included studies demonstrated a low risk of bias, indicating reliable and reproducible methodologies. This structured approach supports evidence-based evaluation and enhances the validity of findings in forensic and clinical dental research related to age estimation techniques.

## Conclusions

Based on the body of evidence reviewed, Cameriere’s I3M emerges as the most accurate method for estimating age in adolescents and young adults. Comparative analyses consistently show that I3M provides greater reliability than the London Atlas for individuals in the 14- to 22-year age range. Although assessments of root pulp visibility can yield slightly higher accuracy among those aged 15 to 22 years, I3M remains a highly dependable approach overall. When compared with other commonly used techniques, including the London Atlas and Demirjian’s stages, I3M demonstrates superior performance. In addition, evaluation using the modified NOS indicates that most dental age estimation studies exhibit low risk of bias, supporting the methodological soundness and validity of the existing research.
